# Description of durum wheat linkage map and comparative sequence analysis of wheat mapped DArT markers with rice and Brachypodium genomes

**DOI:** 10.1186/1471-2156-14-114

**Published:** 2013-12-05

**Authors:** Pasqualina Colasuonno, Mastrangelo Anna Maria, Antonio Blanco, Agata Gadaleta

**Affiliations:** 1 Department of Soil, Plant and Food Sciences, University of Bari “Aldo Moro”, Via Amendola 165/A, Bari 70126, Italy; 2 CRA, Cereal Research Centre, SS16 km 675, Foggia 71122, Italy

**Keywords:** Wheat, DArT marker, Genetic map, Syntheny

## Abstract

**Background:**

The importance of wheat to the world economy, together with progresses in high-throughput next-generation DNA sequencing, have accelerated initiatives of genetic research for wheat improvement. The availability of high density linkage maps is crucial to identify genotype-phenotype associations, but also for anchoring BAC contigs to genetic maps, a strategy followed for sequencing the wheat genome.

**Results:**

Here we report a genetic linkage map in a durum wheat segregating population and the study of mapped DArT markers. The linkage map consists of 126 gSSR, 31 EST-SSR and 351 DArT markers distributed in 24 linkage groups for a total length of 1,272 cM. Through bioinformatic approaches we have analysed 327 DArT clones to reveal their redundancy, syntenic and functional aspects. The DNA sequences of 174 DArT markers were assembled into a non-redundant set of 60 marker clusters. This explained the generation of clusters in very small chromosome regions across genomes. Of these DArT markers, 61 showed highly significant (Expectation < E-10) BLAST similarity to gene sequences in public databases of model species such as *Brachypodium* and rice. Based on sequence alignments, the analysis revealed a mosaic gene conservation, with 54 and 72 genes present in rice and *Brachypodium* species, respectively.

**Conclusions:**

In the present manuscript we provide a detailed DArT markers characterization and the basis for future efforts in durum wheat map comparing.

## Background

Wheat (*Triticum* spp.) is among the most widely grown crops in the world. It is a polyploid species with a nuclear genome characterized by seven homoeologous chromosome groups (A, B and D genomes).

The availability of high density linkage maps is crucial to identify genotype-phenotype associations, but also for anchoring BAC contigs to genetic maps, a strategy followed for sequencing the wheat genome, promoted by the International Wheat Genome Sequencing Consortium (IWGSC, http://www.wheatgenome.org/) [1]. In wheat, large genome size, high percentage of repetitive regions and low level of polymorphism [2] complicated the obtainment of high-resolution genetic maps by molecular markers. The availability of SSR and EST-SSR markers and the development of high-throughput systems, such as diversity array technology (DArT) [3], have overcome these difficulties.

SSR markers, together with expressed sequence tag (EST)-derived SSR markers, have become the markers of choice for cereal genetic mapping because of abundance in plant genomes, high information content, codominant inheritance, and easily detection and reproducibility. In particular EST-SSR markers have been extensively studied in barley [4] and maize [5] facilitating genomic information transfer from related species to wheat.

The DArT system was originally validated for the simple genomes of rice [3] and *Arabidopsis thaliana*[6] and subsequently applied to other crops such as barley [7] and wheat [8]. Deriving from hybridization-based strategy, DArT markers generate whole-genome fingerprints by scoring the presence versus absence of DNA fragments in genomic DNA. Several molecular linkage maps have been developed in wheat, and many of them use DArT markers as anonymous markers [9-13]. The release of the sequence of 2000 wheat DArTs revealed their functional features in relation to their genetic position. The availability of the DArT marker sequences and their abundance in grass genomes changed the role of these markers, proposing them as an optimal tool for comparative genetic mapping or for identification of functional markers.

Comparative mapping is successfully applied in the assembly of sequences or chromosome regions. In this case molecular markers display an important role for their high degree of genetic collinearity across grass genomes because of their common ancestor [14]. Early syntenic analyses were based on RFLP markers, facilitating the evolutionary study of divergence between rice, *Brachypodium* and wheat [15]. The linear order of genes and their strong conservation in large chromosomal regions is described as macro-collinearity. Because of their small and compact genomes, rice and *Brachypodium* were among the first grass genomes to be completely sequenced and were proposed to study the larger and more complex wheat genome as “bridge genomes” [16]. Macro-collinearity relation across rice, *Brachypodium* and wheat genomes was demonstrated at the *Lr34* locus in wheat [17]. However, different studies also have shown that the sequence comparison at the level of individual genes breaks down the macro-collinearity and this was reported as micro-collinearity. At this level it is observed a mosaic gene conservation, with some genes present in both grass species, and some in only one species [16,18,19]. These frequent exceptions in collinearity could depend by translocation, duplication and deletion events [20].

In the present study, we report a complete genetic linkage map developed by crossing two durum wheat varieties characterized by different carotenoid pigment content. The objectives within this topic were: (1) to describe general results of the development of a durum wheat map by gSSR and DArT markers; (2) to extend the sequence analysis of DArT markers in terms of redundancy, comparative and functional analysis; (3) to characterize genetic regions involved in the grain carotenoid content by use of sequenced DArT markers.

## Results

### Genetic linkage map description

**Figure 1:**
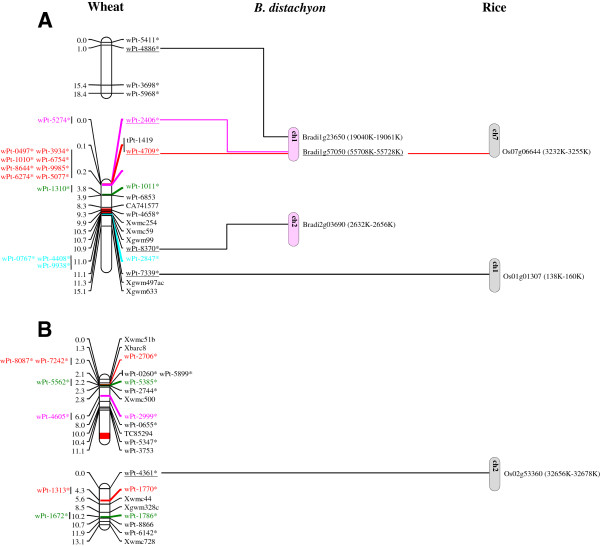
**Genetic map of durum wheat chromosomes of Latino × Primadur population and comparison with *Brachypodium *(pink bars) and rice (grey bars) genomes.** The DArT markers followed by “ * ” correspond to markers for which sequences are available. DArT markers with the same color shared an overlapped sequence, while gSSR and EST-SSR makers are indicating by the black color. Centromeres are indicated by a red square.

To show the distribution of the molecular markers, in particular of DArT markers in relation of their available sequences in durum wheat genome, a genetic linkage map is reported in Figure 1.

The total number of polymorphic markers used to generate the durum linkage map was 546 (141 gSSR, 40 EST-SSR and 365 DArT markers) (Table 1). Moreover five loci corresponding to five genes (two for Glutamine synthetase 2, Psy-1, Psy-2 and Psy-3) were included in the genetic map since they resulted polymorphic between the two parents [21-23]. After the first screening between the two parental lines, the 546 markers were scored in F_2_:F_3_ families.

**Table 1:** Number of markers screened among the parental lines Latino and Primadur and analyzed in the LP population

**Marker class**	**Marker analysed (n.)**	**Marker polymorphic (n.)**	**Marker analysed in the population (n.)**	**Polymorphism (%)**
gSSR	400	141	141	35.3
EST-SSR	450	175	40	38.9
DArT	380	365	365	96.1
Total	1230	681	546	55.4

The complete linkage map consisted of a total of 505 molecular markers (122 gSSR, 32 EST-SSR and 351 DArT markers) distributed in 26 linkage groups (Table 2). The total length of the map accounted 1,272 cM, with an average chromosome length of 90 cM. The total marker number was highest in homoeologous group 7 (total 97 loci), as well as the total map length (259 cM). Homoeologous group 1 had the lowest marker number (total 58 loci) and shortest map length (58 cM).

**Table 2:** **Chromosome assignment, marker distribution, length of linkage groups and marker density in the genetic map constructed with the F**_**2**_**:F**_**3 **_**population Latino Primadur**

**Linkage group**	**SSR**	**DArT**	**Total markers**	**Length (cM)**	**Density (cM/marker)**
1A	6	25	31	33.5	1.1
1B	7	20	27	24.2	0.9
2A	14	29	43	115.3	2.7
2B	12	23	35	142.1	4.1
3A	2	14	16	43.4	2.7
3B	12	49	61	151.8	2.5
4A	7	27	34	75.0	2.2
4B	9	7	16	61.3	3.8
5A	20	6	26	115.6	4.4
5B	21	21	42	132.0	3.1
6A	6	20	26	65.4	2.5
6B	5	46	51	54.1	1.1
7A	16	30	46	136.1	3.0
7B	17	34	51	123.0	2.4
group 1	13	45	58	57.7	1.0
group 2	26	52	78	257.4	3.3
group 3	14	63	77	195.2	2.5
group 4	16	34	50	136.3	2.7
group 5	41	27	68	247.6	3.6
group 6	11	66	77	119.5	1.6
group 7	33	64	97	259.1	2.7
A genome	71	151	222	584.3	2.6
B genome	83	200	283	688.5	2.4
**Total**	154	351	505	1,272.8	2.5
**Average**	11.0	25.1	36.1	90.9	2.6

Forty-one markers (19 SSR, 8 EST-SSR and 14 DArT markers) remained unlinked, probably due to the insufficient coverage of the genome. In fact in some cases we were not able to have a unique linkage group representing one chromosome and no markers were mapped on several bins.

Linkage groups were assigned to chromosomes by comparing markers on the generated map to previously published durum maps. For some chromosome regions (1A, 2A and 6A) the absence of marker anchor loci was replaced by the DArT markers location provided by Triticarte Pty. Ltd.

The 26 linkage groups were assigned to A genome (14) and B genome (12) (Figure 1). The chromosomes 2B, 4A, 5A and 6B were assembled in a single linkage group. The chromosomes 2A and 6A were constructed on 3 linkage groups, while 1A, 1B, 3A, 3B, 4B, 5B, 7A and 7B were represented by two linkage groups. Differences were found between the A and B genomes. B genome had 283 (56%) mapped markers in 688 cM and A genome had 222 (44%) mapped loci in 584 cM (Table 2). The whole 505 mapped loci covered the durum wheat map with a number per chromosome ranging from 16 (3A and 4B) to 61 (3B) (Table 2).

The primer pairs that amplified two or more loci mapped to homoeologous as well as to non-homoeologous sites. The highest number of loci was produced by *Xwmc51*, with two loci mapping on homoeologous group 2 and one locus on chromosome 1B, and by *Xwmc500* with three loci mapping on non-homoeologous chromosomes 4A, 1B and 7B. In five cases SSR markers mapped on the two homoeologous chromosomes: *Xwmc51* and *Xwmc382* primer pairs mapped on homoeologous group 2, *Xgwm132* marker mapped on homoeologous group 6 and *Xcfd6* and *Xcfa2040* loci mapped on homoeologous group 7.

Co-linearity between multi-allelic markers located on homoeologous chromosomes was observed for the group 2 and 7, indeed the two markers of each linkage group maintained the same genetic order. Instead for the *Xgwm132* loci on homoeologous group 6 there was not an evident co-linearity because of the different chromosome location across the arm (*Xgwm132ef-6AL4-0.55-1.00* and *Xgwm132b-6BS5-0.76-1.05*).

The analysis of the marker distributions revealed that there were 56 clusters of DArT markers across the durum wheat genome. Most of them were made of 2 or 3 DArT markers, but others consisted of clusters ranging from 4 to 11 markers at the same locus. In particular, 9 DArT markers clustered in a small region of 0.01 cM on chromosome 6B and 12 DArT markers were grouped in 0.13 cM on chromosome 3B. Other clusters were identified on chromosome 1A (9 DArT markers in 0.04 cm) and 7B (7 DArT markers in 0.15 cM).

The majority of loci in the LP map showed the expected 1:2:1 ratio for co-dominant markers (110 mapped/unmapped gSSR markers) or 3:1 for dominant markers ratio (31 mapped/unmapped gSSR and 365 DArT markers). However, 1 gSSR and 10 DArT markers, out of the 546 markers, presented a significant segregant distortion. Exactly the *Xwmc489*, *wPt-1830*, *wPt-8153*, *wPt-6017*, *wPt-7745-6B*, *wPt-3376-6B* and *wPt-5006* presented distortion in favor of the Primadur allele whereas the other DArT markers (*tPt-7209*, *wPt-8776-1B/2B*, *wPt-0694-2B* and *wPt-9261*) showed distorted segregation in favor of the Latino allele. The loci characterized by segregation distortion were localized on chromosomes 1B, 2B and 6B. These loci were not used in the mapping population.

### Analysis of DArT markers sequences

After scoring 365 DArT markers in the LP population, 351 DArT markers were randomly mapped along chromosomes. Only for 327 mapped DArT markers, sequences were available and they were analysed to search redundant sequences. Of these DArT markers, 201 sequences were not available in the public dataset made of 2000 sequences, already analysed by Marone et al. [10].

Based on high-quality DNA sequence, a total of 174 DArT markers were grouped into two contigs (characterized by overlapping and extended regions) and 58 marker clusters (identical sequences and longer than 400 bp). The remaining 153 DArT clones represented non-redundant sequences since they did not show any overlapping or identical regions with each other. In particular, assembling procedure detected 2 contigs on linkage groups 3B and 6B and 58 marker grouped in clusters. The contig on 3B group has the highest DArT marker number (12 DArTs for a final length of 866 bp), whereas the *wPt-4386-6B* marker grouped into its sequence 9 DArT markers (final length of 637 bp). The majority of sequence DArT clusters shared high identity sequences and were characterized by < 3% mismatches.

No DArT clones sharing high identity sequences appeared to be positioned at different position on the genetic map. This underlines the reproducibility of DArT scores and genetic analysis since markers were in the same allelic phase.

Since grass genomes display a high degree of genetic collinearity because of their common ancestor [14], we used sequences from DArT markers to deduce syntenic relationships between wheat and two model genome species as *Brachypodium* and rice.

The non-redundant set of 153 sequences of mapped DArT markers was searched against the complete non-redundant protein, DNA and EST database (http://genomevolution.org/CoGe/CoGeBlast.pl) of *Brachypodium* and rice genomes to identify segments of collinearity. A compilation of the best BLAST hits from each searched sequence is shown in Additional file 1: Table S1.

The analysis revealed a mosaic gene conservation, with 46 genes (59%) present in both grass species, and 26 and 8 genes only in *Brachypodium* and rice species, respectively.

The identity percentages (*E* value less than e^-10^) for the 72 DArT sequences ranged from 75% (for the multi-loci *wPt-2587-6B* marker), to 100% (for the multi-loci *wPt-5432-3B* marker). Overall, 31 DArT sequences had significant matches to annotated *Brachypodium* proteins (Additional file 1: Table S1).

For the 54 DArT sequences aligned with rice (*Oryza sativa*) genome database, we observed an identity percentages ranging from 74% (*wPt-9277-2A*) to 100% (*wPt-9989-3B*) at *E* value less than e^-10^. Out of the 54 matched sequences, 22 DArT sequences were recognized as annotated rice proteins.

In both analyses, the remaining DArT sequences were classified as uncharacterized proteins or unknown sequences. The comparisons also revealed that among all sequences (annotated, uncharacterized and not matched proteins), 14 *Brachypodium* proteins were also present in the rice EST database, sharing the same functions.

To further identify and validate collinear regions among the grass species, we considered also the EST-SSR or gene markers used for the development of the LP linkage groups as the sequences are available in NCBI. In particular out of the 32 mapped EST markers, 18 ESTs showed high sequence identity with *Brachypodium* genes, whereas 19 markers were aligned with rice genome. Among these 19 markers, 14 EST markers were detected in both genomes with similar annotated functions, identity scores higher than 76% and aligned regions bigger than 150 bp. Only for *TC77302-3A*, *CA694714-4B*, *TC8448-6A* and *BJ236800-6B* markers, we observed a small region of alignment (less than 100 bp) and an unknown function protein in *Brachypodium* and rice genomes. These markers mapped in different loci at *E* value less than e^-10^ with high identity percentages.

Overall syntenic regions were identified on durum wheat chromosomes 3B, 4A, 5B, 6B and 7A, where more than three sequenced DArT were present. Probably due to the gap showed on wheat chromosome 3B, 21 markers (3 SSR and 18 DArT markers) in 22.9 cM matched with two different regions in *Brachypodium* (chromosome 2 and 1) and one rice region (chromosome 1).

Two syntenic regions were also found for wheat chromosome 4A. A region of 54.1 cM (with 3 SSR, 1 EST-SSR and 10 DArT markers) and an overlapped one of 19.2 cM (with 1 SSR, 1 EST-SSR and 19 DArT markers) showed the best hit in the *Brachypodium* sequences of chromosome 5 and 1, while in rice genome it was not possible to identify a well-defined collinearity segment, since it was found on one DArT marker per each chromosome.

An interesting correspondence was found between *Brachypodium* chromosome 1 and a region of 22 cM of durum wheat on chromosome 5B, including a gap on its long arm. A total of 7 markers (2 EST-SSR and 5 DArT markers) had a better alignment with *Brachypodium* genome than rice, in which only two markers were mapped on the same chromosome 3.

A collinear segment was detected on chromosome 3 of *Brachypodium* with 37 cM of wheat segment on chromosome 6B (with 1 SSR, 2 EST-SSR and 42 DArT markers). Most of these DArT sequences had an uncharacterized protein role but showed a better match with *Brachypodium* genome than that of rice.

Remarkable results were detected on wheat chromosome 7A: 33 markers (1 SSR, 9 EST-SSR and 23 DArT markers) were considered in a region of 56.7 cM. Two collinear regions were found in both grass model genomes. The best hits mapped on *Brachypodium* chromosome 1 and rice chromosome 6.

In general, the characterized wheat genomic regions showed more correspondence with the *Brachypodium* genome than that of rice. The high frequency of gene similarity of DArT sequences among both model species provided a good indication that many DArT markers were within rich gene regions.

### Functional analysis of DArT sequences

The availability of DArT sequences can provide opportunities to identify gene loci involved in the carotenoid biosynthesis considering the QTL regions detected (data in [24]). The analysis of DArT markers mapped in all the detected QTL regions allowed to find candidate genes for this trait only on chromosomes 3B (*Xbarc84*-*Xgwm299* and *Xbarc84*-*Xgwm340* intervals).

Since DArT sequences were short in many cases, matches were searched in Cerealsdb database (http://www.cerealsdb.uk.net/) to expand genomic region. The derived longer genomic clones were used as a query for a new BLASTX search against *Brachypodium* and rice protein databases.

The bioinformatic analysis showed that in the QTL region on chromosome 3B, the *wPt-9786*, *wPt-8959*, *wPt-3342* and *wPt-6929* markers matched with genes involved in abscisic acid response, while the *wPt-9989* marker presented homology with gene related to geranylgeranyl diphosphate accumulation. In details, the *wPt-9786* marker highlighted the best hit with both model species for the zinc finger CCCH domain-containing protein, involved in ABA-regulated dormancy [25]. Even the *wPt-8959* marker showed high sequence similarity with *Brachypodium* and rice genome and corresponded to the abscisic stress-ripening protein (*Asr*) gene. This one seems to be involved in some phases of plant development (such as senescence and fruit development) and in responses to abiotic stresses (such as salt and limited light stresses). There are not precise information concerning the biological functions of Asr proteins, but it has been demonstrated a web of interactions around abscisic acid [26].

As confirmed in *Brachypodium* and rice genome, the *wPt-3342* marker belonged to a large transcription factor superfamily related to ABA and auxin responses: the B3 domain-containing proteins [27]. The *wPt-6929* marker had a best hit with the *Brachypodium* genome for G-type lectin S-receptor-like serine/threonine protein kinase, a positive regulator of plant tolerance to salt stress induced by ABA [28].

Instead, the *wPt-9989* marker showed the best hit with the *Brachypodium* and rice protein geranylgeranyl diphosphate reductase. This protein catalyzes the bidirectional reduction of geranylgeranyl diphosphate to phytyl diphosphate, providing phytol for both tocopherol and chlorophyll synthesis [29].

To investigate in details the roles of these selected DArT markers, we performed a comparative matching analysis of DArTs to wheat 61 k microarray platform using the Plant Expression Database (PLEXdb) Blast [30]. PLEXdb identifies the corresponding probe sets on wheat GeneChip with E value < -10 through similarity searches. A total of 6 probe sets were identified as differentially transcribed from the *wPt-0668*, *wPt-0343, wPt-8959*, *wPt-3342, wPt-6929* to *wPt-9989* marker.

Although for the *wPt-0668* and *wPt-0343* markers we observed proteins of unknown function, the analysis in Plexdb revealed that together with *wPt-9989* marker, they showed the highest level of expression in drought stress conditions, starting from root seeds germination phase. The *wPt-8959* marker seemed to be expressed higher in root tissue, while the *wPt-6929* and *wPt-3342* markers showed an invariable expression level in all microarray experiments.

## Discussion

DArT markers are among the most widely used tools in the generation of dense genetic maps. In the present study, we report a 1,272 cM intervarietal genetic map of durum wheat using the LP F_2_:F_3_ segregating population in which SSR and DArT markers were integrated. Latino is a cultivar with high grain yield and adaptability but poor pasta quality parameters, while Primadur is a French variety with a high pigment color. Thus, we analysed the distribution of SSR and DArT markers into LP linkage map and used the DArT sequences to investigate them in collinear studies in *Brachypodium* and rice species.

The primary goal was to provide a molecular and functional map with 122 gSSR, 32 EST-SSR and 351 DArT markers. The LP map confirms other published intervarietal maps according to the positions and orders of commonly mapped markers [9,10,31] and was also used to develop a dense consensus map together with other 5 durum wheat bi-parental maps (Marone et al, 2012b). Efforts were made to minimize the gaps on chromosomes 1B, 2A, 3A, 3B, 4B, 5B, 7A and 7B, but in some cases we were unable to substantially close them for the lack of polymorphisms or for the low marker density, as described in several wheat maps [32,33]. The LP map also shows inter-marker distances higher than 20 cM and chromosome regions 4AS, 4BL and 7AS not covered at all by markers. Lack of genome coverage of homoeologous group 4 was observed in other published wheat maps [9,31,32,34].

The construction of the genetic linkage map indicated a high tendency of DArT markers to have a cluster distribution, determined through marker co-location. We presume that the genomic representations obtained with *PstI* reflect the presence of methylation sites, producing markers in the hypomethylated gene-rich regions [35]. The DArT clones appeared to be located mostly in the telomeric chromosome regions, probably because of the high G + C content of *PstI* sites and of the high recombination rate of these gene-rich regions [9]. The higher density of clusters in one specific region could depend by repeated clones, as confirmed by sequence analysis of clusters sharing highly similar sequences [9].

Based on DArT sequences, we selected contigs or representative markers (from DArT marker clusters) to get a distribution of these markers on *Brachypodium* and rice genomes and to provide information about their functional nature. Our analysis revealed that about one-third of the DArT markers had similar sequences in both model species. As extensively confirmed also by previous studies [17,33], *Brachypodium* resulted the closest wheat relative, allowing the identification of 72 genes in contrast of the 54 genes for rice genome. About 43% and 20% of predicted genes in *Brachypodium* and rice, respectively, were annotated as hypothetical genes. The sequences of *Brachypodium* could be helpful to verify these hypothetical genes in the rice genome.

In light of these results, we identified microsyntenic regions on chromosomes 3B, 4A, 5B, 6B and 7A with bigger size in *Brachypodium* than rice genome. Shatalinal et al. [36], Gu et al. [37], and La Rota and Sorrells [38] revealed the same conserved gene order for these regions starting from large insert clones and not considering microcollinearity through locations with a high number of molecular markers. Many DArT markers (about 80%) were absent in *Brachypodium* genes and about 14% of the rice genes failed in the collinear positions in *Brachypodium*. It is difficult to determine if DArT sequences were lost in the model genomes, if there are alternative isoforms or if they consist of intronic regions, less conserved across grass species. The identity percentages (*E* value less than e^-10^) and the small region size of DArT markers are important characteristics that reduce the gene search into *Brachypodium* and rice species.

A detailed analysis was carried out expanding the DArT sequences on chromosome region 3BL with Chinese Spring genomic clones, when they were available. We focused on this region because it explains a QTL region for yellow pigment [24] with 2 SSR and 14 mapped DArT markers. A high proportion of the DArT clones correspond to sequences involved in the plant response mediated by abscisic acid (ABA) and geranylgeranyl diphosphate metabolism. The ABA corresponds to apocarotenoid molecules that coordinate plant response to water deficit and gene expression, while geranylgeranyl diphosphate is the precursor of all carotenoid components. It is not clear how the ABA -regulated genes (represented by *wPt-9786*, *wPt-8959*, *wPt-3342* and *wPt-6929* markers) and the genes involved in carotenoid biosynthesis (*wPt-6929* marker) could be related.

The analysis in wheat expression database reveals the highest expression level of *wPt-9989* marker in drought conditions, sign of the important function of the carotenoid molecules in stress response. Indeed carotenoids provide precursors for ABA (main apocarotenoid) that plants can accumulate under drought conditions, having effect on plant growth and development. The higher expression level in root tissue of the *wPt-8959* marker (abscisic stress-ripening protein gene) shows its involvement in ABA biosynthesis but not directly in stress conditions. This needs to be better clarified since in barley expression databases the best sequence hit for the *wPt-8959* marker is expressed mostly in roots and after pathogen infection.

## Conclusion

In conclusion, the current analysis provides relevant information on the use of DArT markers as a tool for collinearity studies in grass species and for the identification of genes controlling traits under investigation. Indeed the identification of genes involved in carotenoid biosynthesis makes these markers an important start point for future wheat breeding programs.

## Methods

### Plant material and genotyping

The genetic map obtained from Latino × Primadur (LP) mapping population is reported here in all its parts and integrated with additional molecular markers. Only 5 chromosomes (2A, 3B, 5A, 7A and 7B) were previously published by Blanco et al. [24] for the subsequent QTL analysis in carotenoid pigment content. This is achieved mainly with SSR and EST-SSR markers, and integrated with DArT markers provided by Triticarte Pty. Ltd (Canberra, Australia; http://www.triticarte.com.au) as whole-genome profiling service laboratory.

SSR sequences and PCR conditions were provided and termed from GWM (*Gatersleben Wheat Microsatellite*, [39]), WMC (*Wheat Microsatellite consortium*; [40]; Prasad et al, 2002; [41]), BARC (*Bangladesh Agricultural Research Council*; [42]) and CFA (molecular probes from RFLP). The development of EST-SSR primer pairs was reported by La Rota et al, [43] and available in the public database http://wheat.pw.usda.gov. Primer pairs were chosen in order to represent each microsatellite class and to produce PCR products ranging from 100 to 350 bp in length.

The primer pairs for candidate GS genes mapped on chromosome 2A and 4A were reported by Gadaleta et al. [22]; the primers for candidate Psy1 gene located on chromosome 7A were reported by He et al. [44] and those for candidate Psy3 gene located on chromosome 5A derived by Dibari et al. [21]. The primer pair for candidate Psy2 gene (*forward* TTGGAAATCGAGGTATATGACCT, *reverse* ACTGGACGAACTGGCACAG) located on chromosome 5B derived from a contemporary study not yet published. All the 365 provided DArT markers were indicated with an ID number starting with “wPt” code, followed by a clone number.

### Segregation analysis and map construction

For all of the loci the segregation ratio of 1:2:1 or 3:1 was tested using Chi-squared analysis. The linkage analysis was performed using the Kosambi mapping function within the JoinMap v. 4.0 software [45]. Linkage groups were assembled using a LOD score ≥ 3, after preliminary analysis using LOD score ranging from 2 to 10.

Somers et al. [46], Sourdille et al. [47,48], and Gadaleta et al. [9] previously mapped gSSR markers used as anchor loci and for assigning linkage groups to a particular chromosome. When necessary markers were removed and the order recalculated until a stable and consistent order with the physical position of the markers on chromosome bins was reached.

### Sequence analysis of the DArT clones

The DArT sequences mapped in LP map were kindly obtained by Triticarte Pty. Ltd. The sequences analysis of 327 DArT markers was carried out using the CLC Genomics Workbench software v. 5.5.1 (CLC bio, Muehltal, Germany; limited free version) with the following parameters: nucleotide mismatch cost = 2; insertion = deletion costs =2; length fraction = 0.4; similarity = 0.9; conflict among individual bases resolved by voting for the base with the highest frequency. CLC Genomics Workbench is a program used for assembling similar DArT fragments into longer consensus sequences (known as “contig”). BLASTn homology searches and functional annotation were carried out using COGE terms (http://genomevolution.org/CoGe/CoGeBlast.pl) [49] to compare the non redundant DArT sequences to rice (*Oryza sativa*) and *Brachypodium* genomes. An expectation value (E) of e^-10^ was used as the significant threshold. The DArT markers were assigned to individual grass chromosomes based on their best match to the model genomes. The DArT sequences were also analyzed by BLASTX against NCBI non-redundant protein database (http://www.ncbi.nlm.nih.gov/BLAST). The sequence from DArT markers were also screened in BLASTN search against the Triticeae Repeat Sequence (TREP) database (http://wheat.pw.usda.gov/ITMI/Repeats).

For each DArT analysis EST-SSR or gene sequences were used as control for confirmation of collinear chromosome region.

## Competing interests

The authors declare that they have no competing interests.

## Authors’ contributions

PC performed linkage map development and bioinformatic analysis. AG, AMM and AB contributed to data interpretation and assisted in drafting the manuscript. All authors read and approved the final manuscript.

## Supplementary Material

Additional file 1: Table S1 Genetic and functional features of wheat DArT and EST-SSR markers. All sequences detected in Brachypodium and rice species were selected basing on identity percentages (>70%) and E value (less than e-10). Click here for file
